# Medicinal plants used for traditional veterinary in the Sierras de Córdoba (Argentina): An ethnobotanical comparison with human medicinal uses

**DOI:** 10.1186/1746-4269-7-23

**Published:** 2011-08-04

**Authors:** Gustavo J Martínez, María C Luján

**Affiliations:** 1 Conicet. Museo de Antropología. Universidad Nacional de Córdoba. Hipólito Irigoyen 174. CP 5000 Córdoba, Argentina; 2 Facultad de Ciencias Químicas, Universidad Nacional de Córdoba. Instituto Multidisciplinario de Biología Vegetal (IMBIV-CONICET). CC 495, CP 5000. Córdoba, Argentina

**Keywords:** ethnoveterinary, ethnomedicine, breeders, healers, pharmacopoeia, sierras de Córdoba

## Abstract

**Background:**

This is a first description of the main ethnoveterinary features of the peasants in the Sierras de Córdoba. The aim of this study was to analyze the use of medicinal plants and other traditional therapeutic practices for healing domestic animals and cattle. Our particular goals were to: characterize veterinary ethnobotanical knowledge considering age, gender and role of the specialists; interpret the cultural features of the traditional local veterinary medicine and plant uses associated to it; compare the plants used in traditional veterinary medicine, with those used in human medicine in the same region.

**Methods:**

Fieldwork was carried out as part of an ethnobotanic regional study where 64 informants were interviewed regarding medicinal plants used in veterinary medicine throughout 2001-2010. Based participant observation and open and semi-structured interviews we obtained information on the traditional practices of diagnosis and healing, focusing on the veterinary uses given to plants (part of the plant used, method of preparation and administration). Plants speciemens were collected with the informants and their vernacular and scientific names were registered in a database. Non-parametric statistic was used to evaluate differences in medicinal plant knowledge, use, and valorization by local people. A comparison between traditional veterinary medicine and previous human medicine studies developed in the region was performed by analyzing the percentages of common species and uses, and by considering Sorensen's Similarity Index.

**Results:**

A total of 127 medicinal uses were registered, corresponding to 70 species of plants belonging to 39 botanic families. Veterinary ethnobotanical knowledge was specialized, restricted, in general, to cattle breeders (mainly men) and to a less degree to healers, and was independent of the age of the interviewees. Native plants were mostly used as skin cicatrizants, disinfectants or for treating digestive disorders. Together with a vast repertoire of plant pharmacopoeia, the therapies also involve religious or ritualistic practices and other popular remedies that evidence the influence of traditional Hispanic-European knowledge. Although the traditional veterinary knowledge seems to be similar or else is inlcuded in the local human ethnomedicine, sharing a common group of plants, it has distinct traits originated by a constant assessment of new applications specifically destined to the treatment of animals.

**Conclusions:**

Veterinary medicine is a fountain of relevant vernacular knowledge, a permanent source for testing new applications with valuable ethnobotanical interest. Knowledge on medicinal applications of native plants will allow future validations and tests for new homeopathic or phytotherapeutic preparations.

## 1. Background

Even in developed countries, veterinary care and animal welfare in rural populations is based on ethnomedical veterinary practices, particularly when access to western veterinary products is difficult or too expensive for the local farmer [1]. Traditional veterinary knowledge is comprised by a collection of beliefs and practices regarding animal welfare that involves the use of natural resources (plant and animals) and other materials. This knowledge is generally transmitted orally from generation to generation and, as other traditional beliefs, is currently threatened by technological development, sociocultural changes and environmental changes [2,3]. However, within the industrialized and urban society there is an increasing interest in alternative or complementary medicine which, together with other natural therapies, are based on the use of medicinal plants. Thus, the use of homeopathic and phytotherapeutic remedies in veterinary medicine has gained interest, among other reasons, due to increasing demands on the quality of meat and milk products such as the requirements for producing organic food goods [4].

Orientated towards the documentation of this fast receding traditional knowledge, the description of new resources and sanitary practices and the search for new veterinary drugs, the reviews and databases on veterinary ethnomedicine [5] show that there is an increasing number of scientific contributions on this topic and a vast number of plant taxa have been used for treating animal ailments in Asia, Africa and Central Europe. There are considerably fewer studies on traditional ethnoveterinary in America, and are basically reduced to the treatment of pets in Canada [6-8] the use of natural remedies for domestic animals and breeding in Trinidad and Tobago [9,10] and the traditional knowledge on bovine health in Colombia [11]. In Argentina, the information presented on this subject is mostly folkloric [12-14], and only a few cases present adequate documentation on the remedies used. As an example of the latter, an ethnoveterinary study documented the use of more than 60 plant species by Criollo cattle farmers in the west of the province of Formosa, north of Argentina [15], evidencing the relevance of first-hand information.

Some theoretical approaches to ethno-veterinary studies evidence a remarkable similarity between the therapeutic uses of plants in human and veterinary medicine, using a selection of species associated with a defined cultural pattern and environmental availability. We approach the present study from this same perspective, based on ethnobotanical methodology, to show the interaction between inhabitants, animals and plants used for veterinary purposes within the context of the local cultures of the Córdoba hills. At present, we posses a comprehensive survey of medicinal plants as a part of the ethnobotanical investigations carried out on the Criollo people in this area [16-21]. However, there is no first hand information in veterinary medicinal applications and therefore, the aim of this study was to complete this area of lore. In this sense we propose, as a general aim, to present and interpret the use, role and significance of plants in the veterinary medicine practised by the inhabitants of the Córdoba hills in their social and cultural context. As particular goals we proposed to: characterize the veterinary ethnobotanical knowledge considering age, gender and role of the specialists; interpret the cultural features of the traditional local veterinary medicine and plant uses associated with it; compare the plants used in traditional veterinary medicine with those used in human medicine in the same region.

We hypothesize that there is a significant number of species and medicinal uses still in force and a common cultural matrix of regional traditional practices of veterinary and human medicine, with a remarkable similarity in the application of medicinal plants. It should also be possible to identify an increasing loss of knowledge and practices, mainly associated with the depeasantization of the local dwellers and an underutilization of medicinal plants by the younger generations.

## 2. Methods

### 2.1. Study site and Population

**Figure 1:**
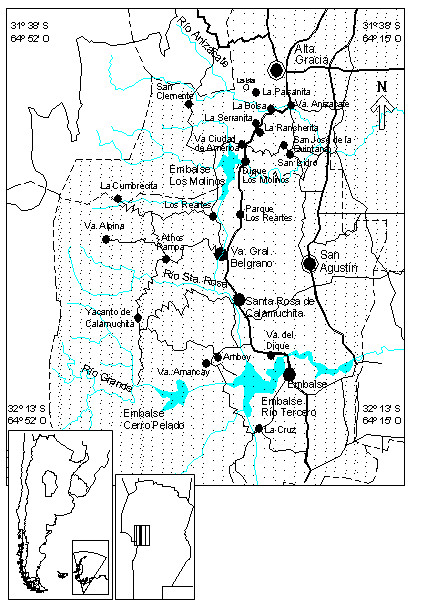
**Study area comprising the valleys of the regions of Calamuchita and Paravachasca in the Sierras de Córdoba, in Central Argentina**.

This study was based on the peasant population of the hills and intermontane valleys of the regions of Calamuchita and Paravachasca (Santa María and Calamuchita Departments) and complemented with surveys carried out in settlements near the town of La Calera, all in the area of the Sierras de Córdoba in Central Argentina, located to the southwest and west of the capital city of Córdoba (Figure 1).

Today, the population of the area is highly heterogeneous, as different groups have settled there. "Criollo" peasants now live side-by-side with immigrants from the last century, as well as city dwellers who have come from Córdoba city and other urban centres. As in other places in Central Argentina the local "Criollo" culture arose as an heritage combination of aborigines and Spaniards or their descendants, after the conquest and foundation of the city of Córdoba in 1573 following a strong Jesuit influence. Between the 19^th ^and 20th Centuries, the European influence, mainly Spanish and Italian, was reinforced as a consequence of the immigration waves. As a consecuence of this historical process, traditional indigenous knowledge, still relative to medicine was practically invisibilized, if not extinct, remaining almost vestigial in their contributions to the current local peasant culture. This study was particularly performed with the native population of farmers and livestock, mainly goat and cattle smallholders. Depending on the economy of the households, cows and horses, mainly, and also goats, sheeps and dogs, receive attention and are treated by traditional veterinary medicine. Although in recent decades become in a progressive depeasantization process, their main economical income are still based on activities such as breeding and selling cattle, tourism-linked services (like horse rental, selling regional products, medicinal herbs, sausages and cheese); recently, in many cases they are also wage earners. Due to environmental shrinkage (related to the advance of monoculture, increasing urbanization, bush fires and loss of native forests), the availability and thus the reliance on the use of plant resources has been significantly reduced among the native local people. The multiple origin of their knowledge, a synthesis between vestigious indigenous lore and European beliefs (traced back to the time of the Spanish Conquest in the 16th Century and reinforced by European immigrants in the last two centuries), explains the similarity to folk medicine found in other Argentinean regions, and the likeness of features found in the traditional Hispanic-European medicine. Also, previous ethnobotanical studies in human medicine carried out in this region describe the validity and entrenchment of traditional healings and folk medicine, mentioning the use of more than 190 medicinal species, most of them natives [17,21].

The climate in the region is mainly semi-humid in summer with maximum temperatures ranging between 28-36°C, and semi-dry in winter with minimum temperatures of 8-14°C. The annual rainfall varies between 700-900 mm with values decreasing to the west and increasing with altitude [22]. The flora comprises the 'Espinal' province and the 'Chaco Serrano' district belonging to the 'Chaco' province, with vegetation alternating between xerophytic plants, shrubs and high pastures. The most frequently found plants are *Prosopis alba*, *Prosopis nigra*, *Aspidosperma quebracho-blanco*, *Celtis tala, Celtis iguanaea, Acacia caven, Geoffraea decorticans, Lithraea molleoides, Zanthoxylum coco, Kageneckia lanceolata*. Among the aromatic and medicinal shrubs, there is a predominance of *Lippia turbinata, Aloysia gratissima, Mintosthacys mollis, Baccharis crispa *and *Baccharis articulata *[23,24].

### 2.2. Data collection and analysis

Fieldwork was carried out as part of an ethnobotanic regional study in which 64 informants (35 women and 29 men) were interviewed about medicinal plants used in veterinary medicine during 2001-2004, 2006 and 2010. The age of the informants ranged from 26 to 88 (X = 61.6 ± 12.8 years). Based on techniques commonly used in ethnobiology and ethnography -as participant observation and open and semi-structured interviews- [25,26], we obtained information on the traditional practices of diagnosis and healing, focusing on the veterinary uses given to plants (part of the plant used, method of preparation and administration, etc.). Interviews were recorded on tapes and registered in field notebooks. Plants speciemens were collected in the company of the informants and their vernacular names were registered. They were then identified by the authors, and herbarium specimens were deposited in the herbarium of the Facultad de Ciencias Agropecuarias, Universidad Nacional de Córdoba under the acronym ACOR, and in the herbarium of the Botanical Museum (IMBIV), acronym CORD. All the information was systematized in a database of medicinal plants which comprises medicinal uses in human diseases obtained in a larger ethnobotanical study developed in the region. Over 45 hours of taped records are deposited at the first author's address. Before being interviewed the peasants were briefed on the research project and its academic objectives. Conversations with specialists and inhabitants were based on a common objective: to increase knowledge regarding natural remedies and develop educational materials of local interest, as suggested in the guidelines of the International Society of Ethnobiology Code of Ethics [27].

Non-parametric statistic was used to evaluate differences in medicinal plant knowledge, use, and valorization by local people using INFOSTAT software [28]. U-Mann Whitney and Kruskal-Wallis tests were used to compare differences between gender and occupation of the informants, respectively (p < 0.05). The Spearman rank correlation was used to analyze medicinal plant knowledge and uses in relation to age (p < 0.05). These tests were the most appropriate because the data did not have normal distribution. A comparison between traditional veterinary medicine and previous human medicine studies developed in the region [17,21] was performed by analyzing the percentages of common species and uses, and by considering Sorensen's Similarity Index.

## 3. Results and discussion

### 3.1. Ethnobotanical knowledge

Regarding ethnobotanical knowledge, 42 informants (65% from a total of 64) knew at least one application in veterinary medicine. An average of 2,95 ± 4,16 (Mean ± S.D.) medicinal uses were mentioned (with a maximum of 20 uses per informant), or 4.97 ± 4.37 when only considering people who cited at least one medicinal use. These highly reduced and variable values in the amount of medicinal uses, especially compared to those mentioned for human medicine as stated ahead, evidence a heterogenous knowledge restricted to certain informers. There are significant differences in the number of uses mentioned between genders (Table 1), with a greater number of applications mentioned by men (Mann-Whitney test, p < 0.05). This table also shows that there are significant differences in the knowledge of medicinal plants according to the occupation of the interviewees (Kruskal-Wallis test, p = 0.01), with most uses described by cattle breeders, followed by livestock workers and healers. This shows that veterinary ethnomedicine knowledge is strictly related to people working with livestick in first place, and to a second degree to healing practices, which in many cases do not only use plants but also symbolic-ritualistic practices like "curing by word" or "by footprints", as described in more detail below. However, most of the interviewed healers were specialized in human medicine, and although some did specialize in animals, very few treated both humans and animals.

There is no correlation between the total number of medicinal plants known and used by the informants and their age (Spearman correlation test, r = -0.03, p > 0.5). It is noticeable that, unlike what is usually evidenced in ethnobotanical studies, veterinary knowledge is not restricted to or more relevant in elderly people in this study area. However, in this case it was found to be restricted to people specialized in livestock.

**Table 1:** Medicinal plant knowledge (n° uses): differences between the gender and occupation of the informants

Gender	N	Mean	S.D.				W (Mann-Whitney)	p
Male	19	4.24	4.48				1107.5	0.021
Female	35	1.89	3.60					

**Occupation**	**N**	**Mean**	**S.D.**	**Ranks**	*		**H (Kruskal-Wallis)**	**p**

Cattle breeder	15	6.27	5.90	45.57	A		10.54	0.01
Healer and breeder	3	4.00	4.58	38.33	A	B		
Healer	9	1.89	2.71	28.06		B		
Other occupations	37	1.78	2.75	27.81		B		

(*) Different letters indicate significant differences (p < 0.05)

### 3.2. Floristic composition and medical applications

A total of 127 medicinal uses corresponding to 70 plant species with veterinary importance belonging to 39 botanical families were gathered and documented in this research.

Table 2 lists the plant species in alphabetical order according to their Latin name. Each plant is presented with its corresponding botanical identity, herbarium sample number, local name, and medical applications, indicating the part used, form of preparation and mode of administration, Specific uses or recipes are indicate in cases where the application is referred to a particular type of animal. Finally, it details identical or similar uses in traditional human medicine recorded in the same region.

**Table 2:** The medicinal plant uses in traditional veterinary medicine of the Sierras de Córdoba. (*) Veterinary medicinal use coincide with human medicinal use

Species (Family) (Voucher number)	Local name	Application	Plant part use Way of preparation and administration	Recipes
*Acacia aroma *Gillies ex Hook. & Arn. (Fabaceae) (AMP 2046)	tusca	wounds and injuries (*)	Aerial part/decoction/washes	Wash the wound with white soap before the treatment.
*Acacia caven *(Molina) Molina var *caven *(Fabaceae) (AMP 1851)	espinillo o aromito	wounds and injuries (*)	Aerial part/decoction/washes	A decoction is prepared alone or with "moradillo", and applies after washing with white soap.
*Acalypha communis *Müll. Arg. (Euphorbiaceae) (AMP 2215)	albahaquilla del campo, curabichera	sores and ulcers (*)	Leaves/decoction/washes	
*Allium sativum *L. (Liliaceae)	ajo	wounds and injuries (*)	Bulb/fat creams/external	Prepare a cream with beeswax and a few cloves of "ajo" and apply to the udder and injures of animals.
		intestinal parasites(*)	Bulb/milled/beverage	Chop four cloves of "ajo" and placed in 1 liter of water and 1 tablespoon of creosote, and is applied in one shot.
		animal and insect bites(*)	Bulb/milled/beverage	Prepare milk and cooking oil with "ajo" and gives drink to the dogs affected by snakebites
*Aloe saponaria *Haw. (Asphodelaceae) (AMP 2105)	aloe de vera, aloe vera, penca aloe	treatment of cattle castrated	Mucilage/direct application/external	
		wounds and injuries(*)	Mucilage/direct application/external	Applied in the "mataduras", sores or injuries of the back
*Aloysia gratissim*a (Gillies & Hook. Ex Hook.) Tronc. var. *gratissima *(Verbenaceae) (AMP 2069)	palo amarillo	wounds and injuries(*)	Leaves/decoction/washes	Apply a decoction of "palo amarillo" alone or with "duraznillo", "doradilla", "cebacaballo" and "manzanilla".
		treatment of retained placenta	Aerial part/decoction/beverage	Combined with "liguilla"; specially for goats.
*Anemia tomentosa *(Savigny) Sw. (Schizaceae) (AMP 1830)	doradilla	wounds and injuries(*)	Fronds/decoction/washes	Apply a decoction of "palo amarillo" alone or with "duraznillo", "doradilla", "cebacaballo" and "manzanilla".
*Aristolochia argentina *Griseb. (Aristolochiaceae) (AMP 2200)	charrúa	muscle pains(*)	Aerial part/alcoholic macerate/friction and massage	It applies to air blows or others cold diseases. Prepare an alcoholic macerate of "charrúa", "ruda macho y hembra", "alcanfor" and "lavanda".
*Artemisia *sp. (Asteraceae) (S/d)	ajenjo chileno	wounds and injuries	whole plant/decoction/washes	
*Baccharis crispa *Spreng. (Asteraceae) (AMP 2058)	carqueja	stomach and abdominal pains(*)	aerial part/decocciòn/beverage	
*Calvatia cyathiformis *(Bosc.) Morgan (Lycoperdaceae) (2229 CORD)	polvillo del diablo, hongo del diablo	wounds and injuries(*)	spores/direct application/topical	Used when the fungus oxidize taking a brown color
		myiasis	spores/direct application/topical	
*Capsella bursa-pastoris *(L.) Medik. (Brassicaceae) (AMP 2076)	bolsa de pastor	breastfeeding, care of the udder	Whole plant/milled/food intake	Mix with food to enhance the udders and to get turgid and pink teats.
*Capsicum annum *L. (Solanaceae)	pimiento (pimentón)	treatment of cattle castrated	fruit/milled/external	
*Celtis ehrenbergiana *(Klotzsch) Liebm. (Celtidaceae) (AMP 2006; AMP 2008)	tala	distemper	bark/smoke bath/inhalation	Is incinerated on lighted coals with sulfur stick.
*Cestrum parqui *L'Hér. (Solanaceae) (AMP 2072)	duraznillo negro	wounds and injuries(**)	leaves/decoction/washes	Apply a decoction of "palo amarillo" alone or with "duraznillo", "doradilla", "cebacaballo" and "manzanilla".
		parasites	leaves/decoction/beverage	It applies to treat Gasterophyllus spp. parasitosis preparing a decoction of "yerba mate", leaves of "duraznillo" and two tablespoons of creosote per liter. Another recipe suggests grinding "duraznillo" with salt in a one liter bottle of water.
		treatment of retained placenta	leaves/decoction/beverage	Applied to cattle
		distemper	root/decoction/beverage	It applies with creosote.
*Chenopodium ambrosioides *L. (Chenopodiaceae) (AMP 2170)	paico	indigestion ("empacho") (*)	whole plant/decoction/beverage	With the root of "quiebraarado".
*Chenopodium *aff. *murale *L. (Chenopodiaceae) (AMP 1979)	quina	wounds and injuries(*)	aerial part/decoction/compresses	Prepare a decoction with 20 g of "quina" and passed through the wounds with a cotton swab.
*Chenopodium album *L. (Chenopodiaceae) (AMP 2243)	quina	wounds and injuries(*)	aerial part/decoction/compresses	Prepare a decoction with 20 g of "quina" and passed through the wounds with a cotton swab.
*Cichorium intybus *L. (Asteraceae) (AMP 1978)	achicoria	bowel purgative	aerial part/food intake	
*Citrus limon *L. (Rutaceae)	limón	ocular diseases	fruit/washes	Wash the affected eye with a rag soaked with water, lemon, salt, crushed leaves of "llanten" and mucilage of the floral bracts of "Santa Lucia".
*Clematis montevidensis *Spreng. var. *montevidensis *(Ranunculaceae) (AMP 2070)	barba de indio, cabello de angel, aloconte	distemper	fruits/smoke bath/inhalation	Burn the fruits with rags, sugar and some drops of creosote and shake the animal to inhale the smoke, to eliminate distemper.
*Colletia spinosissima *J.F.Gmel. (Rhamnaceae) (AMP 1896)	tola tola, barba de indio	wounds and injuries	aerial part/decoction/washes	
*Commelina erecta *L. var. *erecta*(Commelinaceae) (AMP 1981)	Santa Lucía	ocular diseases(*)	mucilage/topical application	Wash the affected eye with a rag soaked with water, lemon, salt, crushed leaves of "llanten" and mucilage of the floral bracts of "Santa Lucia".
*Conyza bonariensis *(L.) Cornquist var. *bonariensis *(Asteraceae) (AMP 2038)	yerba carnicera	diarrhea	whole plant/decoction/beverage	
*Croton subpannosus *Müll. Arg.ex Griseb. (Euphorbiaceae) (AMP 1959)	pulmonaria	bronchial and lung diseases	aerial part/infusion/beverage	With honey
*Cucurbita *spp. [*Cucurbita maxima *Duchesne ssp. *maxima*; *Cucurbita *sp.] (Cucurbitaceae) (AMP 2278; AMP 2284)	zapallo	treatment of retained placenta	seed/decoction/beverage	With salt and "liguilla de chañar" (a shot of a liter per day).
*Ephedra ochreata *Miers (Ephedraceae) (AMP 2146)	tramontana, pico de loro	hits or inflammations(*)	aerial part/cooked/external	Plant fragments are fried in pork fat and applied in the joints of animals and in the "sobrehueso"
*Ephedra triandra *Tul. emend. J. H. Hunz. (Ephedraceae) (AMP 2214)	tramontana, pico de loro	hits or inflammations(*)	aerial part/alcoholic macerate/friction and massage	With "jarilla" and "guayacán".
		hits or inflammations (*)	aerial part/cooked/external	Plant fragments are fried in pork fat and applied in the joints of animals and in the "sobrehueso"
*Equisetum giganteum *L. (Equisetaceae) (AMP 2123)	cola de caballo	kidney diseases(*)	aerial part/infusion o decoction/beverage	
Cfr. *Eryngium *sp. (Apiaceae) (Indet.)	bolo	urinary disorders (difficulty urinating)	flowers/decoction/beverage	
*Eucalyptus cinerea *F.v. Muell. (Myrtaceae) (AMP 2129)	eucaliptus medicinal	distemper(*)	leaves/decoction/inhalation	
*Gaillardia megapotamica *var. *scabiosoides *(Arn. ex DC.) Baker (Asteraceae) (AMP 1846)	topasaire	wounds and injuries(*)	flowers/macerated in oil/frictions	Inflorescences are added in oil burning car, and exposed to the sun for a week.
*Heimia salicifolia *(Kunth) Link (Lythraceae) (AMP 2020)	quiebraarado	hits or inflammations(*)	aerial part/decoction/compresses	
		ocular diseases	root/decoction/eye bath	Prepare a decoction of root of "quiebraarado", root of "cepacaballo" and applies cold as an eye bath
		indigestion ("empacho") (*)	root/decoction/beverage	With "paico"
		diarrhea(*)	root/decoction/beverage	With leaves of "guayacán"
		treatment of cattle castrated	aerial part/washes	
*Ilex paraguariensis *A. St.-Hil.var. *paraguariensis *(Aquifoliaceae)	yerba mate	parasitosis	aerial part/decoction/beverage	It applies to treat "bicho del cuajo" (*Gasterophilus *parasitosis) preparing a decoction of "yerba mate", leaves of "duraznillo" and two tablespoons of creosote per liter.
		distemper	aerial part/decoction/beverage	Give to drink a beverage of creozota, cooking oil and mate.
		intestinal parasites	aerial part/decoction/beverage	Prepare 1 l of mate with salt, creosote and edible oil to treat bug rennet (*Gasterophilus *parasite).
		diarrhea	aerial part/decoction/beverage	To cattle
*Jodina rhombifolia *(Hook. & Arn.) Reissek (Santalaceae) (AMP 2179)	sombra de toro, peje	urinary disorders (difficulty urinating)	aerial part	
*Larrea divaricata *Cav. (Zygophyllaceae) (AMP 2217)	jarilla	hits or inflammations(*)	aerial part/alcoholic macerate/friction and massage	With "guayacán" and "tramontana"
		muscle pain(*)	aerial part/decoction/friction and massage	With "jarilla", "ortiga" and salt.
		treatment of retained placenta	aerial part/decoction (with yerba mate)/beverage	
		constipación	aerial part/decoction with yerba mate/beverage	
		kidney diseases(*)	aerial part/alcoholic macerate/friction and massage	Rub the macerate in the back of the animal to relieve kidney pain.
*Lavandula officinalis *var. *angustifolia *(DeGring.) Briq. (Lamiaceae) (AMP 2285)	lavanda o alhucema	itching and irritation(*)	aerial part/friction and massage	Prepare a cream with salt, lime, sulfur, and lavender and apply in areas of eczema with pus.
*Lepidium didymum *L. (Brassicaceae) (AMP 1974)	quimpe	bleeding gums(*)	whole plant/Fricciones	
*Ligaria cuneifolia *(Ruiz & Pav.) Tiegh. (Loranthaceae) (AMP 2222)	liguilla de flor roja	diarrhea	aerial part/decoction/beverage	With root of "quiebraarado" and "guayacán".
		treatment of retained placenta	aerial part/decoction/beverage	Prepare a drink with salt and a tablespoon of cooking oil and ash. It uses a small branch in 2 liters of water. It is also prepared in decoction with pumpkin seeds (one takes 1 l per day) or "palo amarillo"
*Lippia turbinata *Griseb. (Verbenaceae) (AMP 2142)	poleo	stomach and abdominal pains(*)	aerial part/decoction/beverage	It applies to the cure of indigestion in calves
		wounds and injuries	leaves/decoction/washes	
		kidney diseases	whole plant/symbolic action	A symbolic magical cure is applied for kidney disease of horses. They must pass three times above the plant of "poleo".
		mastitis	leaves/decoction/compresses	It applies in the injured udders of goats and cows, cloths with washes of "malva" and "poleo" with salt.
*Malva parviflora *L. (Malvaceae) (AMP 2081)	malva	intestinal colic(*)	leaves/decoction/enema	With cooking oil, white soap and water boiled.
		ocular diseases	leaves/decoction/washes	Prepare a brine wash with water of "malva" and "ruda".
		wounds and injuries(*)	leaves/decoction/compresses	With water boiled of "malva" and "ruda macho" or "ruda hembra".
		mastitis	leaves/decoction/compresses	It applies in the injured udders of goats and cows, cloths with washes of "malva" and "poleo" with salt.
*Malva sylvestris *L. (Malvaceae) (AMP 1924)	malva	intestinal colic(*)	leaves/decoction/enema	With cooking oil, white soap and water boiled.
		ocular diseases(*)	leaves/decoction/washes	Prepare a brine wash with water of "malva" and "ruda"
		wounds and injuries(*)	leaves/decoction/compresses	With water boiled of "malva" and "ruda macho" or "ruda hembra".
		mastitis	leaves/decoction/compresses	It applies in the injured udders of goats and cows, cloths with washes of "malva" and "poleo" with salt.
*Malvastrum coromandelianum *(L.) Garke ssp. *coromandelianum* (Malvaceae) (AMP 2151)	yerba del potro	wounds and injuries(*)	whole plant/decoction/washes	
		hits or inflammations(*)	aerial part/decoction/compresses	
*Marrubium vulgare *L. (Lamiaceae) (AMP 1966)	yerba del sapo	wounds and injuries	aerial part/smeared in oil and heated/poultice	Fry in oil or mix with green oil and apply as a poultice on infected wounds or animal bites to relieve.
*Matricaria recutita *L. (Asteraceae) (M-ACOR 45)	manzanilla	wounds and injuries(*)	flowers/decoction/washes	Apply a decoction of "palo amarillo" alone or with "duraznillo", "doradilla", "cebacaballo" and "manzanilla".
		treatment of cattle castrated	aerial part/infusion/washes	With warm oil, in crescent moon
*Melia azedarach *L. (Meliaceae) (AMP 2094)	paraíso	fleas(*)	fruits/macerate/baths	The fruits are soaked in water for a day, applying the macerate to dogs with fleas
*Mentha *x *rotundifolia *(L.) Huds. (Lamiaceae) (AMP 2230)	hierba buena	diarrhea(*)	aerial part/decoction/beverage	With peel of "granada"
*Minthostachys verticillata *(Griseb.) Epling (Lamiaceae) (AMP 1894)	peperina	myiasis	leaves/direct application/poultice	It applies in the affected area a handful of crushed or chewed leaves, covering it with horse manure.
		liver diseases	aerial part/decoction/beverage	A decoction with baking soda is given to horses.
*Nicotiana glauca *Graham (Solanaceae) (AMP 1845)	palan palan	wounds and injuries, maturative(*)	leaves/direct application/poultice	To mature spines and wounds
*Nicotiana tabacum *L. (Solanaceae)	tabaco	"tasca"	leaves/beverage	Prepare with "tobacco", milk and cooking oil.
		distemper	leaves/smoke bath/inhalation	
		scabies	leaves/fat creams/external	Apply some of the following mixtures:
				- "Unto sin sal", sulphur, creosote and tobacco, to treat scabies ("sarnilla").
				- lemon, onion and milled tobacco with white soap in warm water.
		parasitosis	leaves/decoction/beverage	It applies to treat "bicho del cuajo" (*Gasterophilus *parasitosis) preparing 1 l of yerba mate with 100 cc of creosote.
*Origanum vulgare *L. (Lamiaceae) (AMP 2132)	orégano	wounds and injuries(*)	aerial part/macerado/washes	Soak for a day, two handfuls of oregano in a half liter of water.
*Phacelia pinnatifida *Griesb. ex Wedd. (Hydrophyllaceae) (AMP 1964)	yerba meona	urinary infection(*)	whole plant/infusion/beverage	Applies when the urine of animals is "charged, heavy, gummy" and prepare an infusion of 6 or 7 plants in a bucket of water.
*Plantago major *L. (Plantaginaceae) (AMP 1940)	llantén liso, llantén grande	hits or inflammations	leaves/smeared in oil and heated/poultice	It applies in internal hits or "tumors" when a horse suffers a heavy blow
		wounds and injuries(*)	leaves/decoction/washes	
		ocular diseases	mucilage/topical application	Wash the affected eye with a rag soaked with water, lemon, salt, crushed leaves of "llanten" and mucilage of the floral bracts of "Santa Lucia".
		bowel purgative	infloerscence and fruits/Food intake	
*Porlieria microphylla *(Baill.) Descole, O'Donell & Lourteig (Zygophyllaceae) (AMP 1941)	guayacán	hits or inflammations	aerial part/alcoholic macerate/friction and massage	With "jarilla" and "tramontana"
		diarrea(*)	aerial part/decoction/beverage	With peel of "Granada" and ember or sugar. With root of "quiebra-arado" and "liga roja".
*Populus *sp. (Salicaceae) (s/d)	álamo	wounds and injuries	leaves/decoction/washes	
*Punica granatum *L. (Punicaceae) (AMP 2294)	granada	diarrhea(*)	fruit peel/decoction/beverage	Alone or with "hierba Buena" or with "guayacán".
*Ruta chalepensis *L. (Rutaceae) (AMP 2244)	ruda macho	intestinal parasites(*)	aerial part/alcoholic macerate/beverage	
		musculoskeletal disorders sprains(*)	aerial part/macerated in kerosene/friction and massage	
		ocular diseases	leaves/decoction/washes	Prepare a brine wash with water of "malva" and "ruda".
		wounds and injuries	aerial part/decoction/compresses	With wáter boiled of "malva" and "ruda macho" or "ruda hembra".
*Ruta graveolens *L. (s/d) (Rutaceae)	ruda hembra	ocular diseases	leaves/decoction/washes	Prepare a brine wash with water of "malva" and "ruda"
		wounds and injuries	aerial part/decoction/compresses	With wáter boiled of "malva" and "ruda macho" or "ruda hembra".
*Salix alba *L. (Salicaceae) (AMP 2121)	sauce alamo	ocular diseases	stem/ashes, burnt/within the eye	Burn a small stem, grind a coal, and blown it with a cartridge of a leaf in the eye of the animal, to make it blink
*Salix fragilis *L. (Salicaceae) (s/d)	mimbre	ocular diseases	tallos/ashes, burnt/within the eye	See recipe in *S. alba*
*Sambucus australis *Cham. & Schltdl. (Adoxaceae) (AMP 2152)	saúco	wounds and injuries	washes	
*Schinus fasciculatus *(Griseb.) I.M. Johnst. (Anacardiaceae) (AMP 2088)	moradillo	treatment of retained placenta	aerial part/decoction/beverage	In cattles
		hits or inflammations(*)	resin ("essence")/direct application/friction and massage	
		wounds and injuries	leaves/decoction/washes	A decoction is prepared with "espinillo", and applies after washing with white soap.
*Sida rhombifolia *L. (Malvaceae) (AMP 2248)	yerba del potro	hits or inflammations(*)	aerial part/decoction/compresses	
		wounds and injuries	whole plant/decoction/washes	The plant is milled and boiled and then prepares a sort of cream that is applied as a bandage to the animal maimed, crippled or injured by thorns
*Tanacetum parthenium *(L.) Sch. Bip. (Asteraceae) (AMP 2156)	altamisa	wounds and injuries	aerial part/decoction/washes	With "altamisa" and "quina".
		animal and insect bites(*)	aerial part/milled/external	It is applied in snake bites with milk and oil, or fried in oil and tobacco.
		treatment of retained placenta	leaves/decoction/beverage	A decoction with "altamisa", flowers of "manzanilla", "moishcurt" (not identified) and three spoon of cooking oil.
*Tripodanthus flagellaris *(Cham. & Schltdl.) Tiegh. (Loranthaceae) (AMP 1970)	liguilla de flor amarilla	treatment of retained placenta(*)	aerial part/infusion o decoction/beverage	With salt and seeds of "zapallo" (a shot of a liter per day).
*Trixis divaricata *(Kunth) Spreng. ssp. *discolor *(D. Don) Katinas (Asteraceae) (AMP 1955)	contrayerba	diarrhea(*)	aerial part/decoction/beverage	
		stomach and abdominal pains(*)	root/decoction/beverage	To treat "empacho" of calfs and goats.
		Fever	root/decoction/beverage	To treat calfs
		distemper(*)	aerial part/smoke bath/inhalation	A smoke bath of "contrayerba" with a few drops of creosote, and dry chicken manure is applied.
		sores and ulcers(*)	aerial part/decoction/washes	To treat varicose ulcers
		wounds and injuries	aerial part/decoction/washes	
*Urtica urens *L. (Urticaceae) (AMP 2210)	ortiga, ortiga negra	muscle pain(*)	aerial part/decoction/friction and massage	With "jarilla", "ortiga" and salt.
*Verbascum thapsus *L. (Scrophulariaceae) (AMP 1841)	gordolobo	stomach and abdominal pains	--	To treat "empacho" of calfs.
*Verbesina encelioides *(Cav.) Benth. et Hook. f. ex A. Gray (Asteraceae) (AMP 2292)	Santa María	wounds and injuries(*)	aerial part/decoction/washes	
*Xanthium spinosum *L. var. *spinosum *(Asteraceae) (AMP 1864)	cepacaballo	ocular diseases(*)	root/decoction/eye bath	Prepare a decoction of root of "quiebraarado", root of "cepacaballo" and applies cold as an eye bath.
		wounds and injuries	root/decoction/washes	Apply a decoction of "palo amarillo", "duraznillo", "doradilla", "cepacaballo" and "manzanilla".
*Zea mays *L. (Poaceae) (M-ACOR 47)	maíz	distemper	leaves, inflorescence/others/external	Cut the tips of the ears of dogs and then put a collar made of cobs around the neck. A smoke bath of burned cobs is applied to treat distemper of the animals.
		scabies	Inflorescence/others/external	It spread a cob of corn with burned mineral engine oil and is applied to the affected sheep

**Figure 2:**
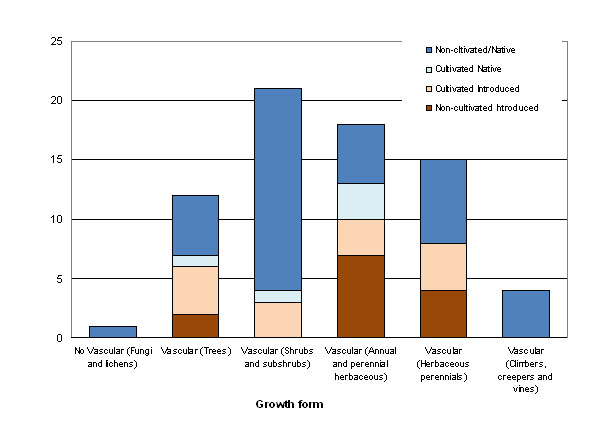
**Number of species used in veterinary medicine according to their growth form and botanical status**.

From a botanical point of view, the species used in veterinary medicine are mostly represented by shrubs, subshrubs and herbaceous species; trees creepers and non vascular forms are less frequent (Figure 2). The use of native wild species is relevant in most cases, as well as the use of some wild introduced species, and both categories surpass the use of cultivated plants. This, together with the wide range of uses given to the native flora of the area, reveals the excellent knowledge and integration peasants have of their surroundings, as well as their great capacity to satisfy their own therapeutic requirements using local plant resources.

**Figure 3:**
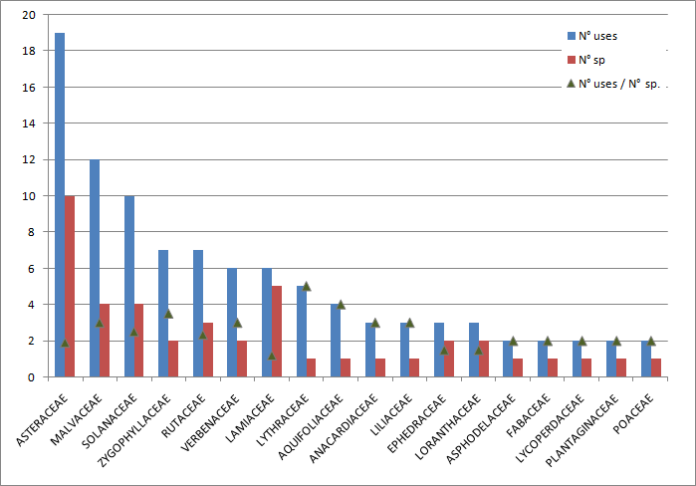
**Botanical families with the most number of uses and species cited for veterinary medicine**. (Families represented have N°uses/N°sp. values greater than 1).

The Asteraceae family is the most commonly used and species-rich family in this study and also the most representative in the medicinal flora of the Province of Cordoba [29]. It is followed by Lamiaceae (by their number of species), and Malvaceae, Solanaceae, Zygophyllaceae, Rutaceae and Verbenaceae (according to their number of uses and species) (Figure 3). Considering the ratio between the number of uses and number of species (N° uses/N° sp.), the Aquifoliaceae and Lythraceae families head the list with *Ilex paraguariensis *and *Heimia salicifolia*, respectively. As in other human ethnomedicine studies we carried out in the region, a significant proportion of these medicinal species are aromatic, especially those belonging to the Lamiaceae, Rutaceae and Verbenaceae families, or have known active substances, as in the case of Solanaceae [18-20]. Moreover, according to Moerman *et al. *[30], Asteracaeae, Lamiaceae, Malvaceae and Solanaceae are some of the most intensely used families for human medicine in different regions of the world. The results obtained in this regional study, would allow extending Moerman's hypothesis of an existence of a global pattern of human knowledge and selection of medicinal plants to the field of veterinary medicine.

Among the species with the greatest number of medical applications we find "contrayerba" (*Trixis divaricata *ssp. *discolor*) with six applications; "duraznillo" (*Cestrum parqui*), "quiebrarado" (*Heimia salicifolia*), "yerba mate" (*Ilex paraguariensis*), "jarilla" (*Larrea divaricata*), "poleo" (*Lippia turbinata*), "malva" (*Malva parviflora*, *Malva sylvestris*), "tabaco" (*Nicotiana tabacum*), "llantén" (*Plantago major*) and "ruda" (*Ruta chalepensis*), each of which have four applications; and finally "ajo" (*Allium sativum*) and "altamisa" (*Tanacetum parthenium*) with three different uses.

Based on the consensus of the interviewed subjects, the following applications were the most popular: the use of "ligas" (*Ligaria cuneifolia *and *Tripodanthus flagellaris*) for treating placental retention, the use of "polvillo del diablo" (*Calvatia cyathiformis *and probably other undocumented Gasteromycetes) and "espinillo" (*Acacia caven*) for healing wounds and sores and the symbolical application of "maíz" (*Zea mays*) for treating animal distemper.

Although this study is not part of a research program on bioactive compounds, there are phytochemical references on some of the plants listed in this article, especially in those with greater consensus of use or with major number of medicinal uses. For example, *Calvatia cyathiformis *has been reported as a good edible species in Britain, North America and Nigeria; in human medicine it is often used as a haemostatic and is also known to inhibit the formation of tumours [31]. Furthermore, pharmaceutical research has led to the isolation of several steroids from these fungi [32-34]. Antiproliferation, inmunomodulator activity and induction of apoptosis has been described for *Ligaria cuneifolia *[35,36]. Although its oxytoxic effects have not been corroborated, it could be related to its high concentration of tyramine that induces toxicity and high blood pressure [37]. The use of *Acacia caven *as an antiseptic, cicatrizant and vulnerary has been described in different ethnomedical studies and a great variety of known active components and substances have been identified in this species [38,39]. However, to the present day studies on its biological activity have only corroborated its effects on a few fungi [40]. Likewise, as mentioned in the review of Goleniowski et al. [40], biological activity has been detected in extracts from plants with less consensus of use among the inhabitants, but that are used in traditional recipes. For example, antibiotic activity has been described for *Aloysia gratissima *and *Lippia turbinata *(used for treating wounds and sores), antifungal activity has been mentioned for *Conyza bonariensis *(used in cases of diarrhoea), and anticancer and antibiotic activity have been described for *Larrea divaricata*.

Regarding the number of species used for differents medicinal applications (Table 3), the most relevant are those used as cicatrizants (for treating wounds and injuries; sores and ulcers; mastitis, and others) followed by plants used for digestive disorders, ostheomuscular disorders and parasitosis.

**Table 3:** Frequency of application of the plants used in traditional veterinary medicine in the Sierras de Córdoba

Medicinal application	Absolute frequency
cicatrizant, disinfectant, antiulcer	43
digestive, stomachic, antidiarrheic, intestinal, hepatic, purging	20
osteomuscular, anti-inflammatory	15
antiparasite (vermifuge and anti-mange)	11
Antiophtalmic	11
pectoral decongestive	9
Oxytoxic	8
diuretic, nephritic	6
anti-poison, treatment of intoxications	3
febrifuge	1

The commonest ways of preparing veterinary plant formulations are decoctions and infusions in water (62%), followed by macerations (6%), direct application (5%) and smoke baths (4%). In general, the whole plants or some of the aerial parts are used (71%). Most preparations are administered externally (60%), either in washes, compresses or by friction, while concoctions for internal use, such as drinks and inhalations, are used to a lesser extent (40%). This contrasts with the most common forms of administration in human medicine for the same study area and population, where 80% of the preparations are for internal use in forms of infusion and decoctions [17,21].

Regarding the plant parts used (Table 4), peasants usually harvest the aerial parts (42% of the cases, 34 spp.) or leaves (27.7% uses, 17 spp.), followed by roots (6.3% uses, 4 spp.) and whole plants (5.4% uses, 4 spp.), which entails a mild to moderate impact on the conservation of these resources. In general, the inhabitants only use the aerial parts of native medicinal species like *Minthostachys verticillata, Trixis divaricata *subsp. *discolor, Baccharis crispa *and *Equisetum giganteum *that are prioritized for conservation according to a study conducted at regional level [41], using the roots only in a few special cases (*T. divaricata*). Furthermore, in some cases (*Minthostachys verticillata*, for example) the local people take particular care when harvesting specimens.

**Table 4:** Plant part used in veterinary medicine ordered by their decreasing values of frequency of use

Plant part	% of uses
Aerial part	42.0
Leaves	27.7
Roots	6.3
Whole plant	5.4
Flowers	3.5
Fruits and seeds	3.5
Bulb	2.7
Spores	1.8
Mucilage	1.8
Bark	0.9
Others	4.4

### 3.3. Other remedies

In addition to the aforementioned plants, many other preparations, concotions and applications involving the most varied ingredients are also used for therapeutic practices. These are described in Table 5, together with a list of ailments (with their vernacular names) that afflict the animals.

**Table 5:** Common folk diseases and treatment practices in traditional veterinary medicine in the Sierras de Córdoba

Diseases	Traditional therapeutical practices
**A) Diseases and treatments related to animal reproduction (gestation, pregnancy, animal birth and breeding)**	

Retained placenta	- Wipe the hindquarter of the animal with wood ash
	- Hang up the rest of the umbilical cord of the spine bone of a dead animal smeared in oil, in order to drain and make a counterweight
Abscesses (Mastitis, inflammation of the udder)	- "Unto sin sal" (Unsalted fat; (Fat soft belly of pork) air dried.
	- Melted fat of a cow
	- Beeswax and garlic

**B) Digestive diseases**	

"Empacho" (indigestion) and diarrhea	- Milk serum curd with salt
	- Draw a cross with cow's milk on the back of foals or calves

**C) Respiratory diseases**	

"Moquillo" (Distemper)	- Mark the face of the horse, drawing a muzzle with kerosene or fat
	- Smoke bath with rubber, and chicken manure incinerated
	- Smoke bath with creosote and a jute bag or rags incinerated
	- Smoke bath with cobs of corn and rags incinerated
	- Puncturing the sinuses in horses with a sharp cane
	- Cut of the ears dogs and leave bleed; then placing a necklace of seven (or an odd number) of burned corn cobs, leaving him in the neck until it heals
	- Cut of the ears of the horse
	- Incision below the "carretilla" (in the region of the jugular vein) of horses
	- Pour one tablespoon of cooking oil in each ear of the horse
	- Break a raw egg on the forehead of the animal

**D) Diseases of the skin and hair**	

Pimples and boils	- Poultice of manure of chicken (*Gallus gallus*) and turkey, fat iguana (*Tupinambis *spp.), and bread crumb with milk
Wounds and injuries	- Burned mineral engine oil
	- Sugar
	- Human stool ("defecation of a christian")
	- Honey
	- White soap
"Mataduras" (Sores) (Healing of wounds on the back of horses)	- Ointment shoes (prefference color like the hair affected animal)
	- Lime
	- Copper sulfate
	- Oil
	- White Liniment
	- "Unto sin sal" (Unsalted fat) with lard
	- Human urine with grated brick
"Capaduras" (Castration) (Scarring of castrated cattle)	- Hot kerosene to remove the "pasmo" (similar to a spasm) due to cold entrance when the animal was castrated
	- Kerosene with salt
	- Ashes with fresh cow manure
	- Preparation of egg yolk, oil and lime
	- Burned mineral engine oil
Hemorrhage	- Spiderweb
	- Paprika
Snake bites (dogs)	- Necklace of braid straw (*Stipa brachychaeta*) wrapped in the neck until it heals
	- Brushstrokes of kerosene in the affected area

**E) Parasitosis**	

"Embichaduras": Myiasis caused by the larvae flies of the "screwworm" (*Cochliomyia hominivorax, *Calliphoridae) and others.	- Hang three leather washers of a male animal, to treat females and, conversely, three female leather washers to treat the males
	- Apply to the navel or to affected areas of a newborn calf an implement fleece (like a cotton swab) dipped in creosote, horse manure, and burned mineral engine oil
Scabies: Caused by the mites *Psoroptes bovis*, (Psoroptidae) and *Sarcoptes *sp. (Sarcoptidae).	- Burned mineral engine oil (sheep scab) applied with corncob
	- "Unto sin sal" (Unsalted fat) with tobacco, sulfur and creosote
"Bicho del cuajo" (Bug rennet): Internal parasitosis due to larvae flies of *Gasterophilus *spp. (Gasterophilidae)	- Mate, salt, cooking oil and creosote intake.

**F) Diseases of the senses**	

"Nubes" "Clouds" (Conjunctivitis)	- Sugar in the inner of the eye (Equine conjunctivitis)
	- Washings with human urine and salt (Equine conjunctivitis)
	- Instill blood of dog ticks (Ovine Conjunctivitis)
	Practice a small incision in the lid of the eye as a cross-shaped
Hits on the eyes	- Sugar

**G) Diseases of the urinary ways**	

"Sarro en la verija" (Tartar in urinary ways) (Anuria due to urinary infections)	- Place the horse under a stream of water and a knife in the genital area, and then hit three kicks until urine

**H) Musculoskeletal diseases**	

Musculoskeletal pain	- Friction with fat of iguana (*Tupinambis *spp), of lion (*Puma concolor*) or of chicken (*Gallus gallus*)

**I) Poisonings**	

For consumption of "chuscho" (*Nierembergia linariaefolia*)	- Break two eggs in the front and draw a cross
For consumption of "manzanilla silvestre" (*Anthemis cotula*)	- Drill the crease of the swollen abdomen, with a knitting needle
"Tasca" (sound made by horses when hitting the jaws), For consumption of "paletaria" (indet.)	- Tobacco, milk and oil intake
For consumption of "romerillo", "nío" or "mío - mío" (*Baccharis coridifolia*)	- Rubbing the plant on the lips and gums of the animal, as a way of prevention
	- Milk and cooking oil intake, or other astringent drink like "anís" or "mate cocido"
	- Vinegar and starch intake

### 3.4. Intoxication by poisonous plants

An important topic in animal health is the presence of toxic plants. Furthermore, it is also particularly interesting for the discovery of new bioactive natural products [42]. Veterinarians generally believe that intoxication of livestock by consumption is a significant problem in the region, especially for cattle coming from other places. Local animals are also affected, but only in times drought and subsequent shortage of pastures during spring regrowth, or in cases of overgrazing. Likewise, cases of intoxication by the consumption of buds, particularly of "duraznillo" (*Cestrum parqui*), may occur when animals seek shelters or remain in enclosures during prolonged rainy periods. While references on this topic give account of more than a hundred potentially toxic plants in Argentina [43], only a dozen were reported by the local people and documented in this paper. The plants were categorized by the informers according to the frequency of intoxication as follows:

- Very frequent intoxication: "romerillo", "nío", "niyo", "nillo", "miyo", "mío-mío" o "niño" (*Baccharis coridifolia *DC., Asteraceae)

- Frequent intoxication: "cicuta" (*Conium maculatum *L., Apiaceae); "duraznillo negro" (*Cestrum parqui *L'Hér., Solanaceae); "chuscho" (*Nierembergia linariaefolia *Graham var. *linariaefolia*, Solanaceae)

- Unusual intoxication: "clavillo" (*Baccharis flabellata *Hook. & Arn.var. *flabellata, *Asteraceae); "cola de quirquincho" (*Huperzia saururus *(Lam.) Trevis., Lycopodiaceae); "chamico" (*Datura ferox *L., Solanaceae); "lagaña de perro" (*Caesalpinia gilliesii *(Wall. Ex Hook.) D. Dietr., Fabaceae); "manzanilla silvestre" (*Anthemis cotula *L., Asteraceae); "paraíso" (*Melia azedarach *L., Meliaceae); "paletaria" (undocumented).

### 3.5. Diagnosis and other folk treatments

When diagnosing an animal's health the inhabitants consider their "countenance", particularly when they appear sad or have drooping ears, symptoms that usually indicate illness. A characteristic trait of traditional veterinary therapy is the inclusion of biomedical concepts, humoral or Hippocratic notions and traditional Spanish medical features in its explanation. There are a diversity of criteria associated with different etiologies assigned to the affections of animals that regulate the prescription of remedies. Thus, bleedings and incisions used for treating distemper are practices historically related to Hippocratic-Galenic or humoral medicine, based on the notion of the regulation of body fluids. Similarly, diseases originated by an imbalance due to excessive heat or cold are treated with plants from the opposite category, in other words cold or hot plants, to re-establish the balance. Although references to this therapeutic criterion for animal diseases are not as frequent as for humans, the explanations indicate that the Hippocratic opposite principle is a still valid in traditional veterinary therapy. For example, loss of blood from a castration predisposes animals to getting "cold" with the consequent risk of the animal suffering from "pasmo"; hence, the prescribed treatments are based on rubdowns with oil, ashes, kerosene, and other "hot" ingredients. Likewise, plants like "peperina" (*Minthostachys verticillata*), "contrayerba" (*Trixis divaricata *ssp. *discolor*) and "ruda" (*Ruta chalepensis*) are considered "hot" remedies. Some historical aspects of these principles, together with a detailed analysis of this therapeutic criterion in the traditional medicine of Argentina, particularly in Córdoba, can be found in other studies carried out by our group [16,19-21,44] and other authors [45-49].

As described for popular Spanish medicine [50], and observing its influence in *Criollo *veterinary medicine, the inhabitants of this area also involve Christian symbolism in their therapies, as in the case of applications or incisions made in the shape of a cross to cure eye or digestive affections. In addition to the wide range of plant pharmacopoeia and popular remedies mentioned above, traditional therapies include religious-ritualistic practices involving prayers, formulas and representations regarding planetary influence and other notions, mostly inherited from traditional Hispanic-Christian medicine. In this sense, the inhabitants assiduously use the following types of treatments:

a) Healings by "rastro" (footprints): they involve printing animal footprints or "rastros" on soft ground or soil, and then cutting out with a spade and usually turning them round. This is used for treating "embichaduras" or myasis, including the navels of newborn animals.

b) Healings by word or prayers: "empachos" (digestive affections), "nervios" (ostheomuscular disorders, sprains and wrenches), the "bicho del cuajo" (*Gasterophilus intestinalis *or horse bot fly), "embichaduras" (myasis), and other afflictions are healed by word. "Nervios" (originated by drafts, bad movements or missteps) are cured by word using a glass of water with wheat for three days. Diagnosis and treatment involve asking the number of years the animal has and throwing 9 grains of wheat into the water while praying and saying the number of affected nerves. This treatment can also be performed at a distance, by praying. This method is also used for cow or horse myasis and the treatment of calf navels, but not on dogs as it could cause the specialist to loose it's power. Cures by word are generally performed by a specialist with a "secret" prayer that is usually learnt on Good Fridays and involves counting down the numbers that represent the nerves, worms or other agents that might be affecting the animal For it to be effective, it is crucial to know the animal's name, type/colour of its coat and the affected place, as well as not to see the animal while the therapy lasts for the cure to occur at "a distance". The treatment of "empacho" in calves also involves symbolic actions like making the sign of the cross on the hip of the affected animal.

c) Practices that consider lunar influence: animal castration is generally carried out during a waning moon because they believe that there is less risk of haemorrhage as blood circulation is decreased in this phase. Likewise, this lunar phase is preferred for getting a horse for it to have "a good mouth" that is not "slobbery" and that has a "good rein".

### 3.5. Comparison with medicinal plant uses in traditional human medicine

Several authors claim that ethnomedical practices are largely the same for animals and people, whether in the form of administration of the materia medica, in the materials themselves, or in surgical, mechanical, behavioural, or medical religious practices [2,10,15,51]. Schillhorn van Veen [51], in an historical and current analysis based on the similarities of both types of medicines, suggested the convenience and application of a broader one-medicine concept integrating animal and human medicines, and the need, in a contemporary context, of a more responsive, safe and effective healthcare system that responds to social changes such as the interest in non-conventional medicines. In general terms, and in accordance with these studies, almost all the plants and some of the medicinal applications used for traditional veterinary practices in the "sierras de Córdoba" have also been described in the local popular human medicine, revealing a remarkable similarity. In fact, and regarding the plant parts used and ways of application, there are not many differences between human and animal therapies [15,17,52]. Also, most of the species with the greatest number of medical applications in traditional veterinary medicine (*Trixis divaricata *ssp. *discolor; Larrea divaricata*, *Malva parviflora*, *Malva sylvestris*, *Plantago major*, *Ruta chalepensis *and *Tanacetum parthenium*) coincide with the twenty species with the greatest number of uses in human medicine [17,21].

A deeper analysis, as shown in Table 6, shows that although the veterinary medicine seems a subset of the human medicine, it presents certain distinct features. This is evident when regarding the number of medicinal taxons involved. Although almost all the species used (98.6%) in veterinary medicine are part of the domestic human ethomedicine, the values of the similarity index (S = 0.53) reveal a group of species that are only used in human medicine (probably because it involves a wider spectrum of affections that are irrelevent in animals). This difference increases when considering the number of medicinal uses with only 45.7% common uses, while the rest are exclusively used for treating of animal affections (especially in cases which have no correspondance with human affections such as myasis, udder infection, "tasca"). The first analysis evidences a considereable difference in the medicinal plant corp, which becomes still more evident considering the low value of the index of similarity of uses (S = 0.13). This allows us to hypothesize that the applications in veterinary medicine were originated based on the human medical ethnobotany, followed by particular applications tested specifically for this ambit. These tests required selecting part of the available plant biodiversity, and used it in the quest of applications destined specifically to animal affections.

**Table 6:** Similarities in traditional veterinary and human medicine in the Sierras de Córdoba, according to their number of species and uses

	Human medicine (A)	Veterinary medicine (B)	In common (C) (% Vet. med. inside H. med.)	Sorensen's Similarity Index
				S = 2C/(A+B)
Number of species	190	70	**69 **(98.6%)	**0.53**
Number of uses	754	127	**58 **(45.7%)	**0.13**

## 4. Conclusions

With a total of 70 medicinal species, *Criollo *veterinary medicine is a fountain of relevant vernacular knowledge, a permanent source for testing new applications with valuable ethnobotanical interest. According to the statistical analysis, veterinary ethnobotanical knowledge seems to be generally restricted to livestock specialists -mainly males-, and is not dependent on the age of the interviewees.

A correspondence between the veterinary use and biological activity is observed for some species or practices, and there great variety of native resources still remain unexplored from a pharmacological point of view. However, the knowledge of new medicinal applications for plants will encourage studying the use of new homeopathic or phytotherapeutic preparations, promoting the advancement of alternative medicines and reducing the dependency on pharmaceutical products, all of which entails an increase in the quality and value of animal products in accordance with new market demands.

Although animal and human ethnomedicine adopt similar therapeutic criteria and share the same cultural matrix, there are visible differences. Traditional veterinary medicine seems to be part of and is originated from traditional human medical practices, with trial and error being the main empirical form of establishing new uses. However, and as expressed by the locals, the offer of veterinary pharmaceuticals, a depeasantization process and environmental pressures, have greatly reduced the use of these practices, causing the loss of this knowledge which in turn is immediately related to the transmission of this information between generations and the loss of experience. A methodological diachronic approach would shed more light on this and contribute a deeper view of the results of this study. It would also allow to understand the future perspective and destination of this ethnological knowledge that is highly valuable to the culture and identity of the Criollo peasant community of the sierras de Córdoba. Due to the relevance of native wild plants in veterinary practices, the dissemination of this traditional knowledge in new generations may lead to a revalorization of local plant resources, promoting the conservation of medicinal flora and regional biodiversity that is particularly threatened in the study area. In this sense, it is be important to recover and record medicinal plant uses in veterinary medicine, within an ethnoscientific context as approached in this study.

## Competing interests

The author declares that they have no competing interests.

## Authors' contributions

GM participated in the planning and design of this study. Both GM and CL carried out the field work, the analysis of the information and wrote the manuscript. All authors read and approved the final manuscript.
